# Chemotherapy‐induced cognitive impairment in breast cancer survivors: A systematic review of studies from 2000 to 2021

**DOI:** 10.1002/cnr2.1989

**Published:** 2024-02-13

**Authors:** Omid Amani, Mohammad Ali Mazaheri, Mona Malekzadeh Moghani, Fariba Zarani, Rasool Hamidi Choolabi

**Affiliations:** ^1^ Department of Psychology Shahid Beheshti University Tehran Iran; ^2^ Department of Radiation Oncology Shahid Beheshti University of Medical Sciences Tehran Iran; ^3^ Department of Psychology Ahrar Institute of Technology and Higher Education Rasht Iran

**Keywords:** breast cancer, chemotherapy, cognitive function, systematic review, women

## Abstract

**Background:**

Studies have indicated that apart from enhancing patient survival, chemotherapy has adverse side effects on the psychological, social, and cognitive functions of breast cancer survivors.

**Aims:**

This study was conducted to understand chemotherapy's impact on breast cancer survivors' cognitive functions.

**Methods and Results:**

Our study is a systematic review based on the Preferred Reporting Items for Systematic Reviews and Meta‐Analyses (PRISMA) statement. We searched English databases, including PubMed/MEDLINE, PsycINFO, and Web of Science, and Persian databases, such as Irandoc and Elmnet, using Persian keywords of cancer, breast cancer, chemotherapy, cognitive functions, executive functions, and neuropsychological functions. Two reviewers independently evaluated the full text of the articles according to predefined criteria. Among the 937 available studies, 26 were selected based on the inclusion and exclusion criteria, of which 17 (65%) were longitudinal and 9 (35%) were cross‐sectional. The findings indicated a significant relationship between the use of chemotherapy and cognitive impairments, most notably attention, working and short‐term memory, and executive functions. However, the studies differed in their findings regarding the long‐term persistence of cancer‐related cognitive impairment (CRCI), which could be due to the wide range of tools used, different methods to measure cognitive functions, and the difference in the sample size of the studies.

**Conclusion:**

Chemotherapy, affecting cortical and subcortical brain structures, causes a set of cognitive impairments that can lead to impairments in social responsibility acceptance, daily functioning, and quality of life of women. Therefore, rigorous and extensive research design is required to understand the causes and consequences of CRCI using standardized and sensitive measures of cognitive functions. Specifically, studies comparing the effects of different chemotherapy regimens on cognition and potential mechanisms and/or moderators of CRCI would be instrumental in designing more effective therapy regimens and evaluating the efficacy and cost‐effectiveness of cognitive rehabilitation and supportive care programs.

## 1 INTRODUCTION

Breast cancer is a major health concern for women, as it is the most prevalent type of cancer and the second leading cause of death among them.^1^, ^2^ In Iran, the age‐standardized incidence rate of breast cancer escalated from 18.8 per 100 000 in 1990 to 34.0 per 100 000 in 2019 among females and from 0.2 to 0.3 per 100 000 among males.^3^ Advancements in oncology, surgery, and treatments such as radiotherapy, chemotherapy, and hormone therapy have significantly improved the survival rate of breast cancer patients, with over 90% surviving for 5 years.^4^, ^5^ Nonetheless, these treatments, especially chemotherapy, have adverse effects on various aspects of the well‐being of breast cancer survivors.^6^

One of the most common and debilitating effects is cancer‐related cognitive impairment (CRCI),^7^, ^8^, ^9^ which affects attention, memory, concentration, learning ability, processing speed, language, and executive functions.^10^ CRCI is a term that encompasses the cognitive changes that may occur during or after cancer diagnosis and treatment. In the past, this phenomenon was sometimes referred to as chemo brain or chemo fog,^11^ as it was first observed among women with breast cancer undergoing chemotherapy in the 1980s.^12^ Currently, using these terms is not recommended as they may create an expectation of negative outcomes, potentially influencing patients' treatment decisions.^13^ Moreover, these terms are inaccurate, as they imply that only chemotherapy affects cognitive functions, while evidence suggests that other factors may also contribute to CRCI.^14^, ^15^ Although CRCI is a vast field that encompasses a wide range of cognitive impairment experienced by cancer survivors, regardless of treatment,^16^ the present review will specifically concentrate on the cognitive impairments that occur as a result of chemotherapy.

The prevalence, duration, and severity of CRCI vary widely across studies. Some studies report that 15% to 50% of breast cancer survivors experience CRCI,^17^ while others estimate that more than 75% of them suffer from it.^18^ Moreover, some studies suggest that CRCI is transient and resolves within a year after completing chemotherapy,^19^ but others indicate that it persists for years after diagnosis,^20^ impairing the quality of life of women.^21^ Additionally, some studies detect cognitive decline in survivors,^22^, ^23^ while others do not.^24^, ^25^, ^26^

These inconsistent findings may be due to the complexity of cognitive functions, differences in measurement tools, and the specific characteristics of each study, such as the demographic characteristics of the sample with breast cancer (such as age, stage of cancer, and menopause), the time of diagnosis and treatment, the type of treatment, and research design.^27^, ^28^, ^29^, ^30^ Moreover, the exact causes and mechanisms of CRCI are still unclear,^31^ but several factors have been proposed to explain how cancer and specifically chemotherapy affect cognitive functions. These include increased gray matter atrophy, metabolic disorders, vascular injuries,^32^, ^33^ and accelerated aging processes.^34^

Given the importance of cognitive functions for daily functioning in breast cancer survivors and the contradictions in the literature about their cognitive impairments, a systematic review is needed to provide reliable information for clinical decision‐making. Several systematic reviews and meta‐analyses have been conducted to summarize the existing evidence on the effects of chemotherapy on the cognitive functions of breast cancer survivors. However, these reviews have some limitations that warrant an updated and comprehensive review. For example, some reviews are outdated and do not include recent studies that have used more advanced methods and tools to assess cognitive functions.^35^, ^36^ Some meta‐analyses have shown conflicting results regarding the impact of chemotherapy for breast cancer on cognitive function. While some studies have reported significant and widespread cognitive deficits across multiple domains, one meta‐analysis found minor deficits.^36^ Some reviews only incorporated studies published in English, potentially introducing publication bias and limiting the diversity of evidence.^35^, ^36^, ^37^, ^38^ Moreover, some reviews have used different criteria or definitions to identify CRCI, such as self‐report measures or neuropsychological tests, which may affect the comparability and generalizability of the results.^35^, ^36^, ^37^, ^38^

Therefore, this systematic review aims to provide a comprehensive and up‐to‐date synthesis of the evidence on the specific effects of chemotherapy on the cognitive functions of breast cancer survivors. It will address the following research questions^1^: which domains or aspects of cognitive functions are most affected by chemotherapy?^2^ how long does CRCI last after completing chemotherapy? and^3^ what is the long‐term effect of using chemotherapy on cognitive functions?

Furthermore, our systematic review will serve as a pivotal point of reference, prompting increased attention and concerted efforts from researchers and healthcare professionals to develop and implement interventions, thereby alleviating the challenges associated with CRCI. This encompasses refining clinical practice, fostering multidisciplinary collaboration, enhancing patient support, and encouraging further research to optimize patient care.

## 2 METHODS

The present study was a systematic review based on the Preferred Reporting Items for Systematic Reviews and Meta‐Analyses (PRISMA) statement. We adhered to the PRISMA checklist criteria^39^, ^40^ (see Appendix S1).

### 2.1 Inclusion/exclusion criteria

We used the Population, Intervention, Comparison, Outcome, Study design (PICOS) framework^39^ to define the inclusion criteria for our systematic review. We included studies that met the following criteria: (i) population: women treated for breast cancer; (ii) intervention: studies focusing on the effects of chemotherapy on cognitive functions; (iii) comparator: not restricted; (iv) outcome: cognitive function assessments in breast cancer survivors; and (v) study design: descriptive research, case reports, and cohort studies. The additional inclusion criteria were (i) studies published in both Persian and English languages; (ii) full‐text article accessibility; and (iii) studies published from 2000 to 2021. This time range was chosen considering the significant advances in chemotherapy and cognitive assessment methods over the past two decades, which might have influenced the results of the studies. Consequently, older studies that might not reflect current knowledge and practice were excluded.

The exclusion criteria are as follows: (i) studies on non‐human subjects; (ii) studies based on experimental and interventional methods; (iii) review and meta‐analysis studies; (iv) articles published in conferences and conventions; (v) articles with no accessible full text; and (vi) studies with serious methodological weaknesses or not meeting minimum qualitative criteria based on the PRISMA checklist.

### 2.2 Search strategy and data sources

Our study, conducted between December 2021 and January 2022, focused on studies published in English and Persian on the cognitive functions of women treated for breast cancer. We carried out a literature search of the English studies available in the PubMed/MEDLINE, PsycINFO, and Web of Science databases during 2000–2021 with the keywords of “breast cancer”, “breast malignancy”, and “chemotherapy” in combination with “cognitive” or “cognition”; “executive function”; “neuropsychological”; along with “dysfunction”; “complaints” using search operators and/or. We also searched for papers published in Persian journals with the Persian keywords of “cancer”, “breast cancer”, “chemotherapy”, “cognitive functions”, “executive functions”, and “neuropsychological functions” using search operators or/and without any time constraints up to 2021. The supplementary file provides more details about the search strategies we used (Appendix S2).

### 2.3 Selection and data collection process

We utilized a two‐stage screening process to ensure a rigorous and unbiased selection of studies. At first, two researchers independently assessed the titles and abstracts of the articles. This step was crucial for quickly identifying studies that potentially met our inclusion criteria while excluding irrelevant ones. Afterward, the same researchers conducted a detailed examination of the full texts of the articles that passed the initial screening. This comprehensive review was based on our predefined inclusion and exclusion criteria. Disagreements between researchers at this stage were resolved through discussion or, if necessary, consultation with a third researcher.

### 2.4 Quality assessment

We employed the Strengthening the Reporting of Observational Studies in Epidemiology (STROBE) statement's 22‐item checklist to evaluate the quality of the studies. Previous research has suggested that observational studies published in high‐quality journals have an average of 69% of the STROBE items. Therefore, we set a cut‐off score of 15 (69%) for inclusion and exclusion criteria.^41^, ^42^ This rigorous assessment ensured that only studies with a high methodological standard were included in our review.

### 2.5 Data extraction and synthesis

Data extraction was conducted systematically, with information on author, year of publication, sample characteristics, study design, number of participants, control group characteristics, measurement tools, and types of cognitive impairment being collated. The PICOS tool was utilized for this purpose. For the synthesis of results, we applied a narrative synthesis method. This approach allowed us to comprehensively analyze and compare the findings across the included studies, providing a nuanced understanding of the impact of chemotherapy on cognitive functions in breast cancer survivors.

### 2.6 Classification of cognitive domains

In order to systematically assess and present the cognitive functions affected by chemotherapy in breast cancer survivors, we adopted a comprehensive classification system for cognitive domains. This classification was based on widely recognized cognitive frameworks and was tailored to the specific context of CRCI. The cognitive domains we focused on included: (i) overall cognitive functions; (ii) executive functions; (iii) attention; (iv) memory; and (v) language functions. Each study included in our review was analyzed based on these cognitive domains. The classification allowed for a structured and comprehensive synthesis of the effects of chemotherapy on cognitive functions, facilitating a clearer understanding of the specific cognitive areas impacted.

## 3 RESULTS

### 3.1 Identification and selection of articles

**FIGURE 1:**
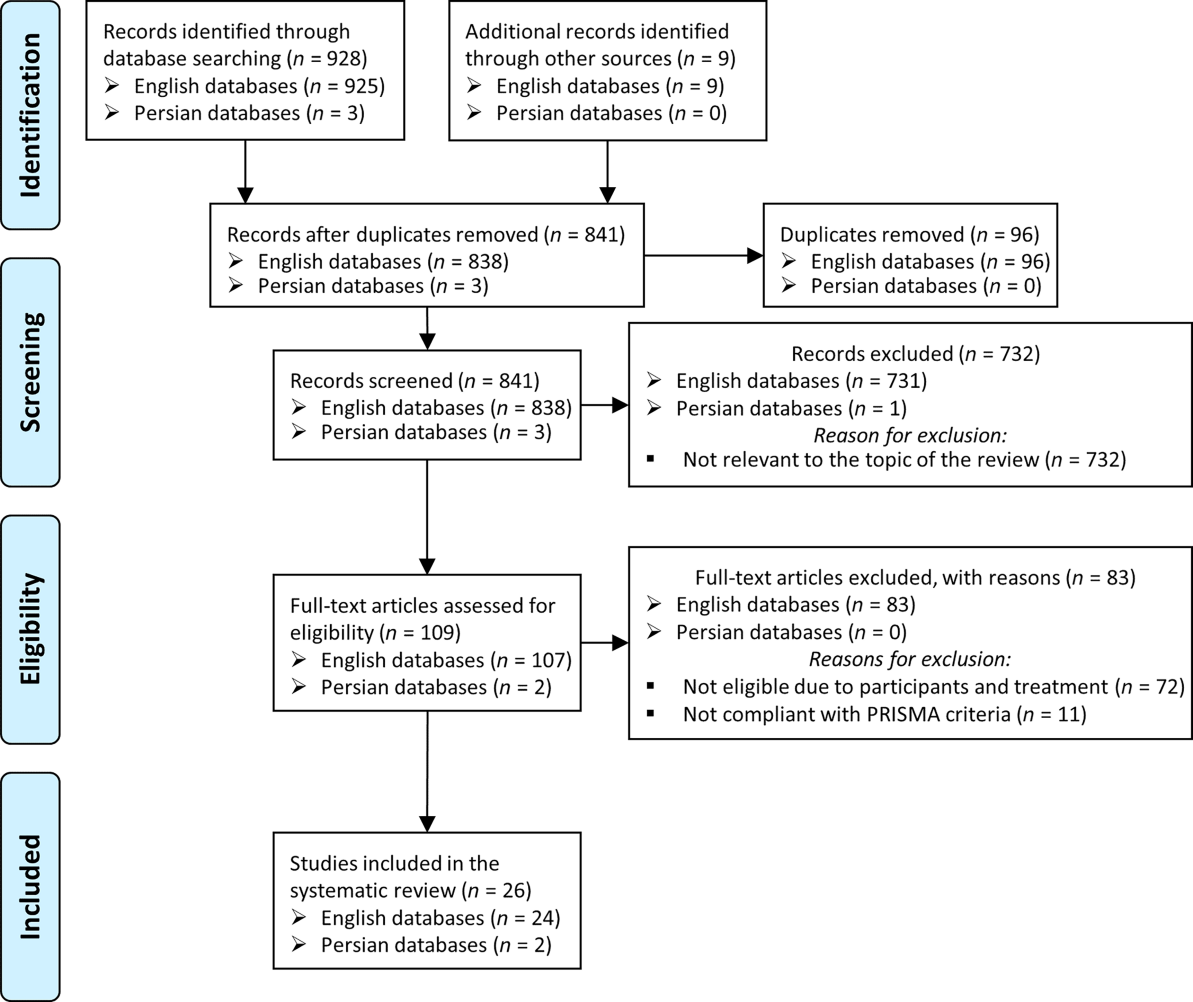
Preferred Reporting Items for Systematic Reviews and Meta‐Analyses (PRISMA) flowchart to illustrate the study search and inclusion process.

Our comprehensive search across electronic databases yielded 937 articles (934 in English and 3 in Persian), initially meeting the PRISMA checklist criteria. We then screened all the articles and excluded 732 studies that did not match the topic of this systematic review, and 96 duplicate studies. In conclusion, 109 studies entered the second screening. Then, we screened all selected papers by the exclusion criteria in terms of the relevance of the abstract to the topic of our study, research design, participants, the treatment used, and the quality of the article. At this stage, we discarded 72 articles due to participant and treatment type issues, and 11 studies for not meeting the minimum criteria related to the PRISMA checklist. Finally, we included 26 studies published from 2004 to 2019 in this systematic review. Figure 1 illustrates the study selection process.

### 3.2 Characteristics of the included studies

Of the 26 selected studies, 17 (65%) were longitudinal, and 9 (35%) were cross‐sectional. Among the longitudinal studies, 10 (38%) evaluated the impact of chemotherapy on cognitive functions at three distinct time points: before treatment, after treatment initiation, and during follow‐up (ranging from one to 6 months). The combined sample size of the included studies was 1629 female breast cancer survivors. The sample sizes ranged from 17^43^ to 196^20^ participants, with participant ages spanning from 44.07^44^ to 72.00^22^ years. The average age was 52.98 years (SD = 7.27). Post‐chemotherapy treatment duration varied from 1 month^45^ to 21 years,^20^ with an average duration of 3.90 years. The studies comprised 19 (73%) with control groups and 7 (27%) without. The control group in 16 studies (61%) included healthy women, in 6 studies (23%) included women with breast cancer treated by other methods, and in 4 studies (16%) included women with breast cancer undergoing other treatment methods. Table 1 details the characteristics and results of each study.

**TABLE 1:** Description of the studies included in this systematic review on chemotherapy's impact on breast cancer survivors' cognitive functions.

Author (year)	Study design	The last course of chemotherapy	Sample size (age)	Reference group size (age)	Measurement tool(s)	Results	Score in the STROBE checklist
Van der Willik et al. (2018)^50^	Causal‐comparative	20 years	166 (64.0 ± 6.7)	1344 healthy peers (57.9 ± 5.2)	MMSE, LDST, WFT, Stroop, PPB, 15 WLT	Decreases in executive function, working memory, verbal memory, attention, and overall cognitive function were observed in breast cancer survivors.	17
Koppelman s et al (2012)^20^	Causal‐comparative	21 years	196 (64.1 ± 6.4)	1509 healthy peers (57.9 ± 5.4)	MMSE, Stroop, WLT, 15‐WLT, PPB	Reductions in attention, working memory, short‐term memory, information processing speed, and psychomotor speed were observed in breast cancer survivors.	15
Nguyen et al. (2013)^22^	Causal‐comparative	10 years	27 (72.0 ± 4.9)	30 healthy peers (72.6 ± 5.5)	MMSE, DS, TMT, COW, BNT, WCST, TMT‐AB, WASI‐II	Declines in the overall cognitive function, attention, working memory, psychomotor speed, executive function, and visuospatial function were observed in breast cancer survivors.	15
Scherwath et al. (2006)^56^	Causal‐comparative	5 years	47 (51.8 ± 8.6)	29 women with breast cancer undergoing chemotherapy (54.6 ± 8.0)	TMT, TAP, WASI‐II, WMS. AVLT	Diminished selective attention, working memory, visuospatial memory, verbal learning, and verbal fluency were observed in breast cancer survivors.	15
Calvio et al. (2010)^62^	Cross‐sectional	3.09 years	122 (44.88 ± 9.51)	113 healthy peers (39.18 ± 11.87)	CSC, PCF, CNSVS	Breast cancer survivors reported symptoms of attention and memory dysfunction that had adverse effects on their work functioning.	
de Ruiter et al. (2012)^43^	Cross‐sectional	10 years	17 (56.5 ± 5.1)	15 breast cancer survivors who did not receive chemotherapy (58.2 ± 5.8)	TOL, Flanker test	Structural changes in the white and gray cortex of the brain and diminished memory, and planning abilities in breast cancer survivors were observed.	15
Mihuta et al. (2016)^57^	Longitudinal	5 years	26 (53.0 ± 6.6)	25 healthy peers (50.4 ± 6.5)	PMT, TMT‐B, Self‐report Measures of Cognitive Function	Decreased performance in event‐based and time‐based prospective memory, attention, processing speed, and cognitive impairments were observed in breast cancer survivors.	15
Ahles et al. (2010)^24^	Longitudinal	Before treatment, 1, 6, and 18 months after treatment began	60 (51.7 ± 7.1)	45 healthy peers (52.9 ± 8.3)	WMS‐III, DS, WAIS‐III, TMT‐B, D‐KEFS, PASAT	Reductions in information processing speed, verbal abilities, working memory, verbal memory, and spatial visual memory were observed in breast cancer survivors.	18
Collins et al. (2013)^28^	Longitudinal	After treatment began and one‐year follow‐up	56 (51.8 ± 7.8)	56 healthy peers (51.3 ± 7.7)	DS, TMT‐A, CNS‐VS, PSI, PASAT, BVMT, HVLT	After treatment began, declines in memory and information processing speed were observed, but in one‐year follow‐ups, only one‐third of the survivors showed these symptoms.	16
Jenkins et al. (2006)^63^	Longitudinal	Before treatment, after treatment began, and one‐year follow‐up	85 (51.5 ± 9.6)	49 healthy peers (51.9 ± 8.9); 43 breast cancer survivors who did not receive chemotherapy (58.9 ± 7.3)	Stroop, DS, WMS, LCT, Cognitive Failures Questionnaires	Immediately after the start and end of the treatment, 80% of the patients reported problems in attention, concentration, and memory. In a one‐year follow‐up, problems significantly decreased, and no significant difference was observed with the control group.	16
Syarif et al. (2019)^49^	Causal‐comparative	6 months	82 (46.01 ± 6.55)	80 healthy peers (39.91 ± 8.49); 81 breast cancer survivors who did not receive chemotherapy (43.06 ± 8.18)	TMT‐B	86.6% of post‐chemotherapy breast cancer survivors reported mild to severe cognitive impairment.	15
Menning et al. (2017)^47^	Longitudinal	Pre‐treatment, post‐treatment, and six‐month follow‐up.	24 (51.2 ± 6.8)	31 healthy peers (51.2 ± 8.2)	ToL, Self‐report Measures of Cognitive Function	There was no difference in the performance of the two groups in the Tower of London test, but in the self‐report measures of cognitive function, the cognitive impairment of breast cancer survivors was more than the reference group.	15
Wefel et al. (2010)^58^	Longitudinal	Before treatment, after treatment began, and one‐year follow‐up	42 (48.8 ± 8.1)	‐	TMT‐A‐B, WAIS‐R, DS	Before starting chemotherapy, 21%, during treatment, 64%, and after stopping treatment, 61% of breast cancer survivors reported problems in attention, memory, learning, and information processing speed.	15
Yao et al. (2017)^52^	Longitudinal	Start of treatment, 1 and 9 months after treatment	28 (45.3 ± 8.5)	20 healthy peers (45.7 ± 11.3)	Stroop, Self‐report Measures of Cognitive Function	No significant difference was observed in the groups between the first and second measurements but comparing the first and third measurements showed more cognitive impairment in breast cancer survivors.	15
Fan et al. (2005)^46^	Longitudinal	Before treatment, after treatment began, and one‐year follow‐up	104 (48.0 ± 2.1)	102 healthy peers (47.1 ± 3.3)	TMT‐A‐B	Cognitive impairment was reported after chemotherapy, and its severity decreased at one‐year follow‐up.	15
McDonald et al. (2012)^65^	Longitudinal	Before treatment, 1 month after treatment began, and one‐year follow‐ups	16 (52.9 ± 8.6)	12 women with breast cancer undergoing chemotherapy (52.7 ± 7.2); 15 healthy peers (50.5 ± 6.0)	N‐back task	During chemotherapy, breast cancer survivors reported brain structural changes along with problems in working memory, but the difference between the two groups decreased after the completion of the treatment.	16
Reid‐Arndt et al. (2010)^21^	Longitudinal	One month after treatment began, follow‐up after 6 months and one year	39 (53.4 ± 9.6)	‐	TMT‐B, Stroop, Self‐reported Cognitive Difficulties	Only 20% of the participants reported problems in memory, processing speed, response inhibition, and speech fluency during the treatment, which was also a determining factor in the patient's quality of life and fatigue.	15
Ruzich et al. (2007)^54^	Longitudinal	Before treatment, during treatment, after completion of treatment, and follow‐up after 6 months	35 (53.0 ± 2.3)	‐	TMT‐AB, TOL, CPT, WCST, WMS, RAVLT	Before treatment, a small proportion of patients had cognitive impairments, but during treatment, patients reported impairments in attention, verbal memory, verbal learning, planning, cognitive flexibility, and processing speed.	15
Quesnel et al. (2009)^48^	Longitudinal	Before treatment, immediately after treatment began, and follow‐up after 3 months	41 (50.3 ± 7.2)	45 healthy peers (47.9 ± 7.4); 40 peers treated with radiation therapy (55.0 ± 7.1)	CFT, RAVLT, TMT, SDMT, DS, VMS, VFT	During treatment, patients reported verbal memory and verbal fluency impairments, and at follow‐up, these impairments were reported more frequently.	15
Von Ah et al. (2009)^66^	Longitudinal	4.6 years	52 (58.2 ± 9.2)	52 healthy peers (59.0 ± 9.0)	AVLT, DS, COWA	Breast cancer survivors had impairments in cognitive functions, such as impairments in memory, verbal learning, and verbal fluency compared to the reference group.	16
Hurria et al. (2006)^64^	longitudinal	Before treatment and 6 months after treatment began	45 (70.0 ± 0.0)	‐	Squire Memory Self‐Rating Questionnaire	Comparing the results obtained after 6 months of chemotherapy indicated diminished in memory function (63%) and impairment in verbal learning (49%) among breast cancer survivors. Also, 27% of patients reported an improvement in memory at the six‐month follow‐up.	15
Castellon et al. (2004)^59^	Cross‐sectional	5 years	53 (48.08 ± 6.3)	19 healthy peers (49.2 ± 6.0)	CVLT, WMS, LM‐WAIS, TMT A‐B, PASAT, Stroop, Self‐report Measures of Cognitive Function	Reductions in verbal learning, visuospatial functioning, and visual memory with psychological distress and fatigue were significantly more in patients who received chemotherapy than breast cancer survivors treated with surgery and subjects without a history of breast cancer.	16
Weis et al. (2009)^51^	longitudinal	After treatment began and a 9‐month follow‐up	90 (49.07 ± 7.6)	‐	TMT A‐B, Go/NoGo	After treatment began, all participants reported cognitive impairments in working memory, the judgment of daily cognitive performance, and sustained attention. In the 9‐month follow‐up, the severity of the impairments decreased, and only 21% of the participants reported cognitive impairments.	15
Jansen et al. (2011)^55^	Longitudinal	Before treatment, 1 week after treatment began, and follow‐up after 6 months	71 (50.03 ± 8.8)	‐	Stroop, GP, AFI	After treatment began, short‐term memory, language, and executive function significantly decreased and remained stable in the long‐term follow‐ups.	15
Amani et al. (2017)^60^	Cross‐sectional	1.7 years	65 (44.07 ± 0.00)	65 healthy peers (42.55 ± 0.00)	N‐back task, TOL	Breast cancer survivors reported impairments in working memory, problem‐solving ability, and planning compared to peers without a history of breast cancer.	15
Dehghani et al. (2018)^45^	Longitudinal	Before treatment and after treatment began	40 (47.0 ± 10.0)	‐	WMS, RAVLT	After receiving chemotherapy, delayed visual memory, immediate auditory memory, and delayed auditory memory decreased, but there was no difference between working memory and everyday memory compared to the pre‐test.	15

Abbreviations: AFI, Attentional Function Index; AVLT, Auditory Verbal Learning Test; BNT, Boston Naming Test; BVMT, Brief Visuospatial Memory Test‐Revised; CVLT, California Verbal Learning Test; CNSVS, Central Nervous System Vital Signs; CSC, Cognitive Symptoms Checklist; CFT, Complex Figure Test; COW, Controlled Oral Word; CPT, Continuous performance task; DS, Digit Span; GP, Grooved Pegboard; HVLT, Hopkins Verbal Learning Test‐Revised; LCT, Letter cancellation task; LDST, Letter Digit Substitution Test; LM‐WAIS, Logical Memory Wechsler Adult Intelligence Scale; MMSE, Mini–Mental State Examination; PASAT, Paced Auditory Serial Addition Task; PCF, Performance‐Based Cognitive Function; PSI, Processing Speed Index; PMT, Prospective memory test; PPT, Purdue Pegboard Test; RAVLT, Rey Auditory Verbal Learning Test; TOL, Tower of London; TMT, Trail Making Test; VFT, Verbal Fluency Test; VMS, Visual Memory Span; WAIS‐III, Wechsler Adult Intelligence Scale‐III; WMS, Wechsler Memory Scale‐Revised; WCST, Wisconsin card sorting task; WFT, Word Fluency Test; 15‐WLT, 15‐Word Learning Test.

### 3.3 Cognitive function domains

The effects of chemotherapy on various cognitive dimensions were explored across the selected studies. These studies employed various outcome measures to evaluate specific cognitive domains. Table 2 summarizes these outcome measures, categorizing them according to the cognitive dimensions they assess.

**TABLE 2:** Main measurement tools for cognitive dimensions.

Cognitive dimension	Main measurement tools	Studies (author‐year)
Overall cognitive functions	MMSE, CFQ, self‐report measures of cognitive functioning, self‐report cognitive difficulties scale	Fan et al. (2005),^46^ Menning et al. (2017),^47^ Nguyen et al. (2013),^22^ Quesnel et al (2009),^48^ Syarif et al. (2019),^49^ Van der Willik et al. (2018),^50^ Weis et al. (2009),^51^ Yao et al. (2017)^52^
Executive functions	WCST, TOL, TMT, PASAT	Ahles et al. (2010),^24^ Amani et al. (2017),^60^ Castellon et al. (2004),^59^ Collins et al. (2013),^28^ de Ruiter et al. (2012),^43^ Fan et al. (2005),^46^ Jansen et al. (2011),^55^ Malekzade et al. (2017),^44^ Menning et al. (2017),^47^ Mihuta et al. (2016),^57^ Nguyen et al. (2013),^22^ Quesnel et al. (2009),^48^ Reid‐Arndt et al. (2010),^21^ Ruzich et al. (2007),^54^ Scherwath et al. (2006),^56^ Syarif et al. (2019),^49^ Wefel et al. (2010)^58^
Attention	Stroop, CSC	Calvio et al. (2010),^62^ Jansen et al. (2011),^55^ Jenkins et al. (2006),^63^ Koppelmans et al. (2012),^20^ Mihuta et al. (2016),^57^ Nguyen et al. (2013),^22^ Ruzich et al. (2007),^54^ Scherwath et al. (2006),^56^ Van der Willik et al. (2018),^50^ Weis et al. (2009),^51^ Wefel et al. (2010),^58^ Yao et al. (2017)^52^
Memory	Computer‐based working memory tests, free recall test, DS, WMS, PMT, VFT, BVMT, N‐back task, Memory Self‐Rating Questionnaire	Ahles et al. (2010),^24^ Calvio et al. (2010),^62^ Castellon et al. (2004),^59^ Collins et al. (2013),^28^ de Ruiter et al. (2012),^43^ Dehghani et al. (2018),^45^ Hurria et al. (2006),^64^ Jansen et al. (2011),^55^ Jenkins et al. (2006),^63^ Koppelmans et al. (2012),^20^ Malekzade et al. (2017),^44^ McDonald et al. (2012),^65^ Mihuta et al. (2016),^57^ Nguyen et al. (2013),^22^ Quesnel et al. (2009),^48^ Reid‐Arndt et al. (2010),^21^ Ruzich et al. (2007),^54^ Scherwath et al. (2006),^56^ Van der Willik et al. (2018),^50^ Von Ah et al. (2009),^66^ Weis et al. (2009),^51^ Wefel et al. (2010)^58^
Language functions	RAVLT, VFT, CVLT, self‐report cognitive difficulties scale	Ahles et al. (2008),^67^ Arndt et al. (2010),^21^ Castellon et al. (2004),^59^ Hurria et al. (2006),^64^ Jansen et al. (2011),^55^ Quesnel et al. (2009),^48^ Reid‐Arndt et al. (2010),^21^ Ruzich et al. (2007),^54^ Scherwath et al. (2006),^56^ Von Ah et al. (2009),^66^ Wefel et al. (2010)^58^

Abbreviations: BVMT, Brief Visuospatial Memory Test‐Revised; CVLT, California Verbal Learning Test; CSC, Cognitive Symptoms Checklist; DS, Digit Span; MMSE, Mini–Mental State Examination; PASAT, Paced Auditory Serial Addition Task; PMT, Prospective memory test; RAVLT, Rey Auditory Verbal Learning Test; TOL, Tower of London; TMT, Trail Making Test; VFT, Verbal Fluency Test; WMS, Wechsler Memory Scale‐Revised; WCST, Wisconsin Card Sorting Task.

#### 3.3.1 Chemotherapy and overall cognitive functions

A total of eight studies investigated the index of cognitive functions using the Mini‐Mental State Examination (MMSE), the Cognitive Fusion Questionnaire (CFQ), the self‐report measures of cognitive functioning, and the self‐report cognitive difficulties scale (see Table 1). The results indicated that chemotherapy is associated with a reduction in overall cognitive function scores in breast cancer survivors post‐treatment.^22^, ^46^, ^47^, ^48^, ^49^, ^50^, ^51^, ^52^

#### 3.3.2 Chemotherapy and executive functions

Executive functions refer to higher‐order cognitive processes that underlie flexible goal‐directed behavior, such as planning and problem‐solving.^44^, ^53^ The evaluation of executive functions was conducted across 17 studies^21^, ^22^, ^24^, ^28^, ^43^, ^44^, ^46^, ^47^, ^48^, ^49^, ^54^, ^55^, ^56^, ^57^, ^58^, ^59^, ^60^ using a range of cognitive tests, including the Wisconsin Card Sorting Test (WCST), the Stroop task, the Tower of London (TOL), the Trail Making Test (TMT), and Paced Auditory Serial Addition Test (PASAT). The findings demonstrated a significant and predictable decline in executive functions among survivors, persisting long‐term post‐treatment (see Table 1). In a study, the executive functions of 60 women who have survived breast cancer were measured at 1, 6, and 18 months after the end of the treatment period. The results showed that the executive function problems started in parallel at the end of the treatment period and persisted at the subsequent follow‐ups.^24^

#### 3.3.3 Chemotherapy and attention

The ability to select and examine a particular aspect of the environment and ignore other aspects is defined as attention, which is considered a crucial factor in memory.^61^ In the studies, we reviewed, attention was mainly measured using the color‐interference Stroop task and Cognitive Symptoms Checklist (CSC). Deficits in attention and simultaneously memory problems have been patients' most prevalent cognitive problems during chemotherapy, and 12 studies confirmed this finding^20^, ^22^, ^50^, ^51^, ^52^, ^54^, ^55^, ^56^, ^57^, ^58^, ^62^, ^63^ (see Table 1).

#### 3.3.4 Chemotherapy and memory

The cognitive ability to store and utilize information, known as memory, is a multifaceted phenomenon that manifests in various forms, such as working memory, short‐term memory, and verbal memory.^61^ To measure memory, several cognitive function measures were utilized. These included computer‐based working memory tests, free recall test, Digit Span (DS), Wechsler Memory Scale‐Revised (WMS), Prospective Memory Test (PMT), Verbal Fluency Test, Brief Visuospatial Memory Test‐Revised (BVMT), N‐back task, and Memory Self‐Rating Questionnaire. The results of 22 studies^20^, ^21^, ^22^, ^24^, ^28^, ^43^, ^44^, ^45^, ^48^, ^50^, ^51^, ^54^, ^55^, ^56^, ^57^, ^58^, ^59^, ^62^, ^63^, ^64^, ^65^, ^66^ showed that chemotherapy use is related to the memory problems of people who have experienced cancer. In this way, working memory dysfunction^20^, ^21^, ^22^, ^24^, ^28^, ^50^, ^51^, ^56^, ^58^, ^60^, ^62^, ^63^, ^64^, ^66^ is the most reported problem, followed by short‐term memory^20^, ^43^, ^55^, ^62^ and verbal memory,^24^, ^48^, ^50^, ^54^ in four studies, visuospatial memory^24^, ^45^, ^59^ in three studies, and past and prospective memory,^57^ respectively, included the most studies (see Table 1).

#### 3.3.5 Chemotherapy and language functions

This domain assesses language‐related abilities, including verbal fluency and learning. language functions were measured using the Rey Auditory Verbal Learning Test (RAVLT), VFT, California Verbal Learning Test (CVLT), and the self‐report cognitive difficulties scale. Results showed a significant decrease in language abilities post‐chemotherapy in dimensions such as verbal learning^54^, ^55^, ^56^, ^58^, ^59^, ^64^, ^66^, ^67^ and verbal fluency^21^, ^48^, ^56^, ^66^ (see Table 1).

## 4 DISCUSSION

In this systematic review, we examined 26 studies to explore the potential link between chemotherapy and cognitive impairments in breast cancer survivors. These studies investigated various cognitive functions using a set of neuropsychological tests and self‐report questionnaires. Consistent with previous systematic reviews, our review suggests a potential correlation between chemotherapy and cognitive impairment,^30^, ^31^ most notably attention,^20^, ^22^, ^50^, ^51^, ^52^, ^54^, ^55^, ^56^, ^57^, ^58^, ^62^, ^63^ working and short‐term memory,^20^, ^21^, ^22^, ^28^, ^43^, ^44^, ^45^, ^48^, ^50^, ^51^, ^54^, ^55^, ^56^, ^58^, ^59^, ^62^, ^63^, ^64^, ^65^, ^66^, ^67^ and executive functions.^21^, ^22^, ^43^, ^44^, ^47^, ^54^, ^55^

In the first section of our results, the reviewed studies indicated a potential connection between chemotherapy and cognitive impairments. The observed impairments span various cognitive domains, such as overall cognitive function, executive functions, retrospective and prospective memory, language functions (including verbal learning and fluency), visual–spatial perception, and processing speeds. Thus, chemotherapy appears to be a potential predictor of these cognitive impairments. The basis for these cognitive deficits may lie in structural changes in the brains of those undergoing chemotherapy.^9^, ^27^, ^59^ Chemotherapy can potentially affect brain regions like the left anterior cingulate, middle frontal gyrus, precuneus, bilateral insula, and left middle frontal gyrus.^30^

However, assessing long‐term cognitive impairment necessitates long‐term follow‐up, a challenge evident in the reviewed longitudinal studies. While certain studies^20^, ^22^, ^50^, ^57^, ^67^ suggest persistent cognitive deficits years after treatment, others^46^, ^51^, ^54^, ^63^ indicate these might be transient. For example, one study of 166 breast cancer survivors who had completed chemotherapy an average of 20 years ago found that most performed poorly on memory and attention tests.^50^ However, another study reported that cognitive impairments were more severe after treatment but showed a decrease in severity during follow‐up assessments.^51^ Similarly, another study found that more than 80% of the survivors reported attention deficits shortly after starting chemotherapy, but these problems reduced in severity after 1 year.^63^

In exploring the factors contributing to cognitive impairments in breast cancer survivors, our review highlights the complexity of attributing these impairments solely to chemotherapy. Some early studies attributed the cognitive changes to aging and noted that older subjects were more affected by chemotherapy. However, later studies controlled for age and found persistent cognitive impairments even 10 years after the treatment.^22^ Some studies also suggested that different types of chemotherapy and drugs had different effects on cognitive functions, but others did not find a significant correlation between the type of chemotherapy agent (such as taxane, anthracycline, 5‐fluorouracil, cyclophosphamide, and methotrexate) and the risk of dementia.^68^ Also, initial explanations considered the cause of the disease and type of cancer as the reason for cognitive impairments, but subsequent studies observed no significant correlation between them.^12^ One study compared verbal fluency among three groups: those treated with high‐dose chemotherapy, those treated with medium‐dose chemotherapy, and healthy peers. After controlling intervening variables such as age, education, intelligence scores, use of tamoxifen, and the last time of treatment (peer‐to‐peer method), the results suggested that verbal fluency could be affected by chemotherapy and that the high‐dose group performed worse than the other two groups.^56^ Another study controlled for education, ethnicity, and menstrual factors and found that breast cancer survivors who underwent chemotherapy reported more verbal impairments than healthy women and breast cancer survivors who did not receive chemotherapy.

In light of these findings, it appears that chemotherapy may be linked to cognitive impairments, but there is no definitive evidence that it is the exclusive or primary cause. Our review of three studies with adequate methodology, sample size, and control of confounding variables found that 94%, 86%, and 61% of their participants, respectively, had cognitive impairments after chemotherapy.^49^, ^58^, ^66^ These impairments affected more than half of the survivors and had a significant impact on their daily functioning. Many of them reported difficulties in performing simple tasks such as cooking, keeping track, paying bills, and finishing them on time. They also experienced deficits in their cognitive abilities, such as memory, multitasking, calculation, and language, which could lead to anxiety, depression, fatigue, and poor quality of life.

The variability in study methodologies, including the range of cognitive function measurement tools and differences in sample sizes, contributes to the challenge of drawing definitive conclusions. The sample sizes in the reviewed studies varied from 16 to 196 participants, and control groups ranged from women with no history of breast cancer to those treated with alternative methods. Thus, the current evidence suggests a complex interplay of factors influencing cognitive function post‐chemotherapy without providing definitive conclusions about the long‐term effects of chemotherapy alone.

Our study's limitations include the limited number of languages, the absence of a meta‐analysis, and the lack of protocol registration. The decision to forego a meta‐analysis was based on several key considerations. First, the included studies exhibit a wide range of designs, methodologies, and statistical analyses, introducing significant heterogeneity and potentially undermining the validity of any pooled estimates. Second, the cognitive assessment tools used in the studies varied, each with its psychometric properties and scales, making it challenging to generate comparable effect sizes. Third, the studies have widely varying sample sizes and age demographics, contributing to heterogeneity and potential bias. Fourth, the comparison groups across studies varied, some comparing against healthy peers and others against breast cancer survivors who did not receive chemotherapy, leading to potential confounding. Fifth, the timing of cognitive assessments relative to chemotherapy treatment varied greatly among studies, affecting the understanding of cognitive trajectory post‐treatment. Given these challenges, we opted for a narrative synthesis to provide a more nuanced understanding of the evidence regarding chemotherapy's impact on cognitive functions in breast cancer survivors.

Despite these limitations, our study's strength lies in its comprehensive examination of cognitive impairments. We recommend future research to investigate the psychological and psychosocial impacts of these impairments. Clinicians and healthcare professionals must pay more attention to CRCI and develop cognitive rehabilitation programs to address these challenges.

## 5 CONCLUSION

Our systematic review provides a comprehensive examination of the existing literature on cognitive impairments in breast cancer survivors post‐chemotherapy. While the review suggests a potential correlation between chemotherapy and cognitive impairments, it also highlights the complexity and multifactorial nature of these impairments. The evidence does not conclusively establish chemotherapy as the sole or primary cause of cognitive deficits. Instead, it points to a complex interplay of factors, including age, type of chemotherapy, and individual patient characteristics. The findings underscore the need for further research to understand the long‐term effects of chemotherapy on cognitive functions and to develop effective interventions. To enhance the well‐being of breast cancer survivors, it is essential to determine the risk factors, mechanisms, and interventions for CRCI and pursue more research on its causes, evaluation, treatment, and prevention.^15^ By doing so, we can improve the diagnosis and management of CRCI and provide better support and care for this population.

## AUTHOR CONTRIBUTIONS

**Omid Amani:** Conceptualization (equal); data curation (equal); formal analysis (equal); investigation (equal); methodology (equal); project administration (equal); resources (equal); supervision (equal); validation (equal); visualization (equal); writing – original draft (equal); writing – review and editing (equal). **Mohammad Ali Mazaheri:** Conceptualization (equal); data curation (equal); formal analysis (equal); investigation (equal); methodology (equal); project administration (equal); resources (equal); software (equal); supervision (equal); validation (equal); visualization (equal); writing – original draft (equal); writing – review and editing (equal). **Mona Malekzadeh Moghani:** Writing – original draft (equal); writing – review and editing (equal). **Fariba Zarani:** Data curation (equal); formal analysis (equal); writing – original draft (equal); writing – review and editing (equal). **Rasool Hamidi Choolabi:** Writing – original draft (equal); writing – review and editing (equal).

## FUNDING INFORMATION

Funding sources played no role in the study design, data collection, data analysis, data interpretation, or report writing.

## CONFLICT OF INTEREST STATEMENT

The authors have stated explicitly that there are no conflicts of interest in connection with this article.

## ETHICS STATEMENT

For this type of study, formal ethics approval is not required.

## Supporting information


**Appendix S1.** Supporting information.

**Appendix S2.** Supporting information.

## Data Availability

The data that support the findings of this study are available from the corresponding author upon reasonable request.
