# Supplementary material for: Chemotherapy‐induced cognitive impairment in breast cancer survivors: A systematic review of studies from 2000 to 2021
Source: Cancer Rep (Hoboken). 2024 Feb 13;7(2):e1989. doi: 10.1002/cnr2.1989 (PMC10864736; doi:10.1002/cnr2.1989)
Supplement: Supplementary file 2 — Appendix S2. Supporting information. [file CNR2-7-e1989-s001.docx]

**Appendix:** Search strategies

Pubmed: 1410

((((((Breast Cancer[Title/Abstract]) OR (breast malignancy [Title/Abstract])) OR (chemotherapy [Title/Abstract])) AND ((((((cognitive [Title/Abstract]) OR (cognition [Title/Abstract])) OR (executive function [Title/Abstract])) OR (neuropsychological [Title/Abstract])) AND (dysfunction [Title/Abstract])) OR (complaints *[Title/Abstract]))

PsycINFO: 315

(TITLE-ABS-KEY (Breast Cancer) OR TITLE-ABS-KEY (breast malignancy) OR  TITLE-ABS-KEY ( chemotherapy)  AND  TITLE-ABS-KEY ( cognitive)  OR  TITLE-ABS-KEY ( cognition)  OR  TITLE-ABS-KEY ( executive function)  OR  TITLE-ABS-KEY ( neuropsychological)  AND  TITLE-ABS-KEY ( dysfunction ) )

Web of Science: 1280

TS=( Breast Cancer OR breast malignancy OR chemotherapy) AND TS=( cognitive OR cognition OR executive function OR neuropsychological) TS=( dysfunction OR complaints)
